# Fruquintinib inhibits VEGF/VEGFR2 axis of choroidal endothelial cells and M1-type macrophages to protect against mouse laser-induced choroidal neovascularization

**DOI:** 10.1038/s41419-020-03222-1

**Published:** 2020-11-27

**Authors:** Xiaojuan Liu, Aisong Guo, Yuanyuan Tu, Wendie Li, Lele Li, Wangrui Liu, Yuanyuan Ju, Yamei Zhou, Aimin Sang, Manhui Zhu

**Affiliations:** 1 Department of Pathogen Biology, Medical College, Nantong University, Nantong, Jiangsu China; 2 Department of Traditional Chinese Medicine, Affiliated Hospital of Nantong University, Nantong, Jiangsu China; 3 Department of Ophthalmology, Lixiang Eye Hospital of Soochow University, Suzhou, Jiangsu China; 4 Department of Ophthalmology, Ningbo Eye Hospital, Ningbo, Zhejiang China; 5 Department of Ophthalmology, Affiliated Hospital of Nantong University, Nantong, Jiangsu China; 6 Department of Neurosurgery, Affiliated Hospital of Youjiang Medical College for Nationalities, Baise, Guangxi China; 7 Medical College, Nantong University, Nantong, Jiangsu China

**Keywords:** Cell migration, Drug regulation, Diseases

## Abstract

Wet age-related macular degeneration, which is characterized by choroidal neovascularization (CNV) and induces obvious vision loss. Vascular endothelial growth factor (VEGF) family member VEGF-A (also named as VEGF) and its receptor VEGFR2 contribute to the pathogenesis of CNV. Choroidal endothelial cells (CECs) secret C–C motif chemokine ligand 2 (CCL2), which attracts macrophages to CNV lesion and promotes macrophage M1 polarization. Accordingly, infiltrating macrophages secret inflammatory cytokines to promote CNV. In vivo, intravitreal injection of fruquintinib (HMPL-013), an antitumor neovascularization drug, alleviated mouse CNV formation without obvious ocular toxicity. Meanwhile, HMPL-013 inhibited VEGF/VEGFR2 binding in CECs and macrophages, as well as macrophage M1 polarization. In vitro, noncontact coculture of human choroidal vascular endothelial cells (HCVECs) and macrophages under hypoxia conditions was established. HMPL-013 downregulated VEGF/VEGFR2/phosphoinositide-3-kinase/protein kinase B (AKT)/nuclear factor kappa B pathway and CCL2 secretion in HCVECs, as well as VEGF/VEGFR2-induced macrophage M1 polarization under hypoxia condition. In addition, HMPL-013 inhibited HCEVC derived CCL2-induced macrophage migration and M1 polarization, along with macrophage M1 polarization-induced HCVECs proliferation, migration, and tube formation. Altogether, HMPL-013 alleviated CNV formation might via breaking detrimental cross talk between CECs and macrophages.

## Introduction

Age-related macular degeneration (AMD), a leading cause of incurable vision loss in the elder people and accounting for 8.7% of all cases of blindness in the developed nations^1^, is categorized into dry and wet types. Dry AMD is featured by multiple drusen deposits and rarely impacts vision, developing not only to geographic atrophy but also to wet AMD, which is characterized by choroidal neovascularization (CNV) and induces obvious vision loss. Vascular endothelial growth factor (VEGF) family members, containing VEGF-A (also named as VEGF), VEGF-B, VEGF, VEGF-D, VEGF-E, and placental growth factor (PGF), promote CNV via binding to their respective receptors vascular endothelial growth factor receptor 1 (VEGFR1), VEGFR2, and VEGFR3. Intravitreal injection of anti-VEGF reagents is deemed to be the optimal treatment for CNV. However, any improvement is accompanied by long-term monthly intravitreal injections and ocular complications, such as endoophthalmitis^2^. Therefore, the searching of cellular and molecular mechanisms of CNV is warranted. Clinical studies have shown that age-related changes in Bruch’s membrane lead to choriocapillaris atrophy, as well as to decreased diffusion of oxygen toward the neuroretina. The resulting outer retina hypoxia may be an important driving force of CNV formation, by stimulating VEGF overexpression via the transcription factor hypoxia-inducible factor 1α (HIF-1α) in the retinal pigment epithelium (RPE) cells, and retinal Muller cells^3^. VEGF binds to its receptor VEGFR2, consequently phosphorylates Tyr1175 inside VEGFR2, finally promotes laser-induced CNV formation in mice^4,5^. Anti-VEGF-A/VEGFR2 or nonspecific small interfering RNA inhibits CNV and attenuates VEGF mRNA expression in a mouse laser-induced CNV model^6^. In addition, resveratrol inhibits HIF-1α accumulation and VEGF secretion induced by cobalt chloride through sirtuin 1 in human RPE cells^7^. In addition, in AMD-relevant models, VEGF/VEGFR2 blockade does not cause retinal atrophy, which is a side effect caused by intraocular injections of VEGF-neutralizing proteins^8^. These studies attract us to investigate the suppression of VEGF/VEGRR2 axis in CNV.

Accumulating study reveals infiltrating macrophages contribute to the progress of CNV. In different environments, macrophages polarize into M1 pro-inflammatory and M2 anti-inflammatory types. M1-type macrophages with pro-inflammatory functions can produce VEGF and promote neovascularization^9^, while it has higher transcript ratio of M1 chemokine C–X–C motif chemokine ligand 11 (CXCL11) to M2 chemokine C–X–C motif chemokine ligand 22 (CXCL22) in advanced AMD maculae compared to the control^10^. Besides, M1-type macrophages secret pro-inflammatory cytokines, such as interleukin-6 (IL-6), tumor necrosis factor-α (TNF-α), and C–C motif chemokine ligand 5 (RANTES) to promote ocular neovascularization^11^. C–C motif chemokine ligand 2 (CCL2) produced and released by choroidal endothelial cells (CECs) draws macrophages with CCL2 receptor CCR2 on the surfaces of macrophages to the site of CNV injury^12^. In addition, CCL2 facilitates macrophage M1 polarization^13^.

Fruquintinib (HMPL-013) is an antitumor neovascularization drug, which belongs to tyrosine kinase inhibitors, acting as a powerful and highly selective inhibitor for all types of VEGFRs, including VEGFR1, VEGFR2, and VEGFR3. The researchers have completed phase III clinical trials of colorectal cancer (CRC), and the results show that in patients with metastatic CRC who have received at least two chemotherapy regimens, oral HMPL-013 significantly improves the overall survival rate of the patients compared to the placebo group^14^. Following China’s priority review of HMPL-013 in September 2017, on September 4, 2018, the National Medical Products Administration granted HMPL-013 for the first global approval for the treatment of progressive CRC^15^. Therefore, the question whether HMPL-013 can alleviate CNV attracts our attention.

Herein, mouse laser-induced CNV and in vitro endothelial cell and macrophage hypoxia models were applied to identify the functions and mechanisms of HMPL-013 on CNV. Our study could supply a potential therapeutic strategy for the treatment of wet AMD.

## Materials and methods

### Mouse laser-induced CNV model and treatment

Nine to 10-week-old male C57BL/6 mice were purchased from the Laboratory Animal Center of Nantong University (Nantong, China). The mouse laser-induced CNV model was constructed, as previous description^16^. At the time of laser photocoagulation, the production of a bubble was regarded as a rupture of the Bruch’s membrane, indicating that the model was successfully established. Photocoagulation spots containing hemorrhage or failing to develop a bubble at the laser site were excluded. The mice were randomly assigned into five groups: normal, CNV 7 d, CNV 7 d + 1 μl of 0.1% dimethyl sulfoxide (DMSO), CNV 7 d + 1 μl of HMPL-013 (Elunate^®^; Chi-Med, China; 5 μg/μl in 0.1% DMSO), and CNV 7 d + 1 μl ranibizumab (RBZ; Lucentis; Genentech Inc.; 10 μg/μl; used as the positive control), which is a recombinant humanized monoclonal antibody fragment binding VEGF-A. In the animal experiments, the investigator responsible for all other experiments except for CNV model construction were blind to the group allocation.

### Analysis of HMPL-013 concentrations in mouse retina/RPE/choroid tissues

The HMPL-013 concentrations in mouse retina/RPE/choroid tissues following HMPL-013 intravitreal injection was measured at 0, 4, 8, 12, 16, 20 24, 28, 32, 36, 40, 44, and 48 h through solid-phase extraction followed by liquid chromatography/tandem mass spectrometry, using a stable-labeled internal HMPL-013 standard ([^14^C] HMPL-013) according to previous description^17^.

### Fundus angiography

Fundus fluorescein angiography (FFA) and indocyanine green angiography (ICGA) in mice were done following previous study^18^. For the grading of CNV leakage, two masked researchers not involved in laser photocoagulation or angiography evaluated the fluorescein angiograms at a single sitting. Grade 0 lesions had no hyperfluorescence. Grade 1 lesions exhibited hyperfluorescence without leakage. Grade 2A lesions exhibited hyperfluorescence in the early or midtransit images and late leakage. Grade 2B lesions showed bright hyperfluorescence in the transit images and late leakage beyond treated areas (grade 2B lesions were defined as clinically significant), as previous description^19^. For each ICGA examination, the entire lesion area was quantitatively measured using the Heidelberg software (Spectralis Acquisition and Viewing Modules; version 3.2, Heidelberg Engineering) by three independent observers. Mean observed values were calculated.

### Choroidal flat mounts and immunofluorescence

Choroidal flat mounts and immunofluorescence were performed according to previous methods^20^. Fluorescein isothiocyanate (FITC)-conjugated isolectin-B4 (IB4; #L2895, Merck, USA), rhodamine-conjugated phalloidin (#R415, Thermo Fisher Scientific, USA), DyLight 488-conjugated p-VEFGR2 (#SPC-1381, Stress Marq Biosciences, USA), Alexa Fluor^®^ 647-conjugated nitric oxide synthase 2 (NOS2; #209027, Abcam, USA), phycoerythrin (PE)-conjugated arginase 1 (Arg1; #IC5868P, R&D, USA) and PE-conjugated anti-F4/80 antibody (#ab105156, Abcam, USA) antibodies, and 4′, 6-diamidino-2-phenylindole (DAPI; #ab228549, Abcam) were used in the immunofluorescence.

### Hematoxylin–eosin stain

On day 7, following euthanasia, the mouse eyes were enucleated and immersion-fixed 10 in 4% PFA for 2 h. After fixation, the eyes were embedded in Tissue-Tek^®^ optimum cutting temperature compound (#4583, Sakura Finetek, Japan), and cross-sectioned on a cryostat vertically through the center of the cornea and optic nerve. Slide of 5 µm thickness was stained with hematoxylin–eosin (HE).

### Terminal deoxynucleotidyl transferase dUTP nick-end labeling

After fixation and permeabilization, the mouse cryosections were incubated with a terminal deoxynucleotidyl transferase dUTP nick-end labeling (TUNEL) reaction mixture (#11684795910, Roche, Switzerland) at 37 °C for 60 min, and DAPI for 5 min at room temperature (RT), and then washed for 30 min in PBS.

### Electroretinography

The mice were dark-adapted over 16 h. Then the mice were anesthetized and their pupils were dilated. Contact lens electrodes were placed on both eyes with a drop of methylcellulose. Full-field electroretinographies (ERGs) were recorded by using the universal testing and electrophysiologic system 2000 (UTAS E-2000, LKC Technologies). The responses were recorded at a gain of 2 k using a notch filter at 60 Hz, and were bandpass filtered between 0.1 and 1500 Hz. In the light-adapted photopic state, with a −1.02 log cds/m^2^ background light (flash intensity) to desensitize the rods and isolate cones, photopic cone responses were recorded in response to a single flash of 0 dB. The amplitude of the a-wave was measured from the baseline to the lowest negative-going voltage, whereas peak b-wave amplitudes were measured from the trough of the a-wave to the highest peak of the positive b-wave.

### Western blot

Proteins of retina–RPE–choroid complex tissues harvested from mice or whole cell lysates were obtained by a protein extraction kit (#PROTTOT, Merck, USA). Protein concentrations were measured by a Bicinchoninic Acid Protein Quantification Assay Kit (#23225, Thermo Fisher Scientific, USA). The samples were adjusted into 40 μg protein content and mixed with a suitable volume of sodium dodecyl sulfate (SDS) sample buffer (#LC2676, Thermo Fisher Scientific), separated in proper SDS gel, and transferred onto polyvinylidene fluoride membranes (#IPVH00010, Merck). A total of 5% nonfat dried milk in Tris-buffered saline with Tween 20 was used to block these membranes. After incubation with primary antibodies overnight at 4 °C, membranes were incubated with secondary antibodies for 2 h at RT. Primary antibodies included VEGF (#ab52917, Abcam), p-VEGFR2 (#2478), VEGFR2 (#2479), p-phosphoinositide-3-kinase (PI3K; #4228, Tyr458 and Tyr199), PI3K (#4292,), p-protein kinase B (AKT) (#9271, Ser473), AKT (#9272), p-P65 (#3033, Ser536), P65 (#8242), C–C motif chemokine receptor 2 (CCR2; #ab203128, Abcam), NOS2 (#178945, Abcam), Arg1 (#PA5-29645, Thermo Fisher Scientific), and glyceraldehyde-3-phosphate dehydrogenase (GAPDH; #9485, Abcam). The secondary antibodies included horseradish peroxidase (HRP)-conjugated goat anti-rabbit antibody (#7074) and HRP-conjugated goat anti-mouse antibody (#7076). The antibodies not mentioned were all purchased from Cell Signaling Technology, USA. Enhanced chemiluminescence (#WBULS0500, Merck) were used to visualize protein bands, and ImageJ (National Institutes of Health, USA) was used to measure average band intensities. The relative protein level of molecules in normal group was normalized to one, as previous description^21^.

### Co-immunoprecipitation

Proteins of mouse retina–RPE–choroid complex tissues from normal, CNV 7 d, CNV 7 d + DMSO, and CNV 7 d + HMPL-013 groups were obtained by the protein extraction kit (Merck) and precleared. The beads coated with p-VEGFR2 or VEGF antibody were incubated with the precleared whole proteins 4 °C for overnight. The beads were washed with cell lysis buffer four times. Finally, the beads were boiled for 10 min. The eluents were analyzed by Western blot with VEGF and p-VEGFR2 antibodies.

### Immunofluorescence on cryosections

VEGF and p-VEGFR2 were examined on 8 μm cryosections (on day 7 after laser photocoagulation). The cryosections were blocked with 1% bovine serum albumin for 4 h at RT, then incubated with VEGF antibody (1:50) and p-VEGFR2 antibody (1:50) at 4 °C overnight. For VEGF and p-VEGFR2 staining, antigen retrieval was obtained through heated water bath at 37 °C for 10 min. Thereafter, the slides were stained with Alexa Fluor 488-conjugated goat anti-rabbit IgG (1:200; #A27034, Thermo Fisher Scientific), Alexa Fluor 546-conjugated goat anti-mouse IgG (1:200; #A11030, Thermo Fisher Scientific), and DAPI (1:500). The photomicrographs were taken by a digital high-sensitivity camera (Hamamatsu, ORCA-ER C4742-95, Japan).

### ELISA

Mouse IL-6 (#RAB0309), TNF-α (#RAB047), and RANTES (#RAB0077) and CCL2 (#RAB0055) ELISA kits were purchased from Merck. The ELISA was done following the manufacturers’ instructions.

### Cell noncontact coculture and treatment

Human choroidal vascular endothelial cells (HCVECS; #36052-03, Celprogen, USA) were cultured in Dulbecco’s Modified Eagle Medium (DMEM) containing 4.5 g/l glucose supplemented with 10% (v/v) FBS, 100 U/ml penicillin, and 100 mg/ml streptomycin. The human macrophages were derived from human peripheral blood mononuclear cells and cultured, as the previous description^22^. The cells were kept at 37 ^°^C in a humidified atmosphere containing 5% CO_2_. HCVECs were cultured in the lower well and macrophages were cultured in the upper well of the transwell plate (#CLS3397, Corning, USA) and verified by STR profiling.

The cells were culture in CO_2_ incubator (#BBD6220, Thermo Fisher Scientific; 1%O_2_, 94%N_2_, and 5%CO_2_) for 24 h (hypoxia group), HMPL-013 (0.05 μmol/l for 24 h), CCL2-neutralizing antibody (#AB-479-NA, R&D Systems, USA; 5 μg/ml for the last 30 min), recombinant human CCL2 protein (#279-MC, R&D Systems; 100 ng/ml for 24 h), geraniin (macrophage polarization modulator; #PHL80994, Merck; 30 μM for the last 2 h), or LPS (macrophage M1-type polarization agonist; *Escherichia coli* LPS, serotype 0127:B8; #L3129; Merck, USA; 2 μg/ml for 24 h).

### Quantitative reverse transcription-PCR

Using Trizol reagent (#15596018, Thermo Fisher Scientific), the total RNA was extracted from macrophages. After quantifying RNA by the GeneQuant pro RNA/DNA Calculator spectrophotometer (Amersham Biosciences, Germany), 100 ng RNA for each sample was reverse transcribed by PrimeScript^®^ RT reagent Kit (#RR047A, Takara, Japan). The transcript of GAPDH was used as a control. Then, aliquots of the cDNA production were acquired by a relative quantitative real-time PCR analysis by SYBR Premix EX Taq^®^ II (#RR820A, Takara) in the ABI Prism 7900 (Applied Bio systems, USA). The primers were designed according to the published nucleotide sequences of IL-6, TNF-α, RANTES, CD206, Arg1, chitinase-like protein 3 (YM1), adhesion G-protein-coupled receptor E1 (F4/80), and GAPDH (accession numbers: NM_031168.2, NM_013693.3, NM_013653.3, NM_008625.2, NM_007482.3, NM_009892.3, NM_001355722.1, and NM_008084.2, respectively). The primers in Table 1 were purchased from Sangon Biotech (Shanghai).

**Table 1:** The sequences of primers used in the study.

Gene name	Sense sequence (5′–3′)	Antisense sequence (5′–3′)
IL-6	GCTCCCTACTTCACAAGTCC	GCAGGTTTGCCGAGRAGATC
TNF-α	AGCCCACGTCGTAGCAAACCACCAA	ACACCCATTCCCTTCACAGAGCAAT
RANTES	TGCCCACGTCAAGGAGTATTTC	AACCCACTTCTTCTCTGGGTTG
CD206	CAGGTGTGGGCTCAGGTAGT	TGTGGTGAGCTGAAAGGTGA
Arg1	CTGGAACCCAGAGAGAGCAT	CTCCTCGAGGCTGTCCTTT
YM1	GGGCATACCTTTATCCTGAG	CCACTGAAGTCATCCATGTC
F4/80	TGTCTGAAGATTCTCAAAACATGGA	TGGAACACCACAAGAAAGTGC
GAPDH	CATCACTGCCACCCAGAAGACTG	ATGCCAGTGAGCTTCCCGTTCAG

### Transwell assay

After the coculture with HCVECs or macrophages for 24 h, the macrophages or HCVECs were collected and seeded into the upper chamber (8 µm) at a density of 1 × 10^5^ cells/well (Corning) with non-serum DMEM medium. The lower chamber was filled with 500 µl DMEM supplemented with 10% FBS. Twelve hours later, the human macrophages or HCVECs on the upper surface of the membrane were removed with a cotton swab. Then, the lower cells were fixed with formaldehyde and stained by crystal violet for 30 min. The number of migrated cells was counted under a microscope.

### 5-Ethynyl-2-deoxyuridine assay

The 5-ethynyl-2-deoxyuridine (EdU) incorporation assay was conducted with an EdU kit (#C10310-1, RiboBio, China) according to the manufacturers’ instructions. The cell nuclei were counterstained with DAPI. The EdU-positive ratio was calculated as the cells. The number of cells was counted using Image-Pro Plus software (Media Cybernetics, USA).

### Tube formation assay

HCVECs tube formation was analyzed using extracellular matrigel vessel-like formation assay. Firstly, precooled growth factor reduced matrigel (#354230, Corning) was coated in the bottom of 48-well plate, 300 µl/well and incubated in a humidified atmosphere of 5% CO_2_ at 37 ^°^C for 1 h. After coculture with macrophages for 24 h, HCVECs were harvest and 1 × 10^5^ cells/well was seeded to the coated plate. Then, 20 ng/ml human VEGF-A recombinant protein (#ab55566, Abcam) was added to each well after cells were seeded in triplicates. After culture for 24 h, tube formation was visualized by Olympus microscope (Japan), and total tube length were analyzed using Image-Pro Plus software.

### Statistical analysis

The data were shown as mean ± SEM. Statistical analysis was performed by the one-way ANOVA followed by Tukey’s test. *P* < 0.05 was considered statistically significant. Analysis was done by statistical software SPSS 15.0.

## Results

### HMPL-013 mitigates mouse laser-induced CNV formation

**Fig. 1:**
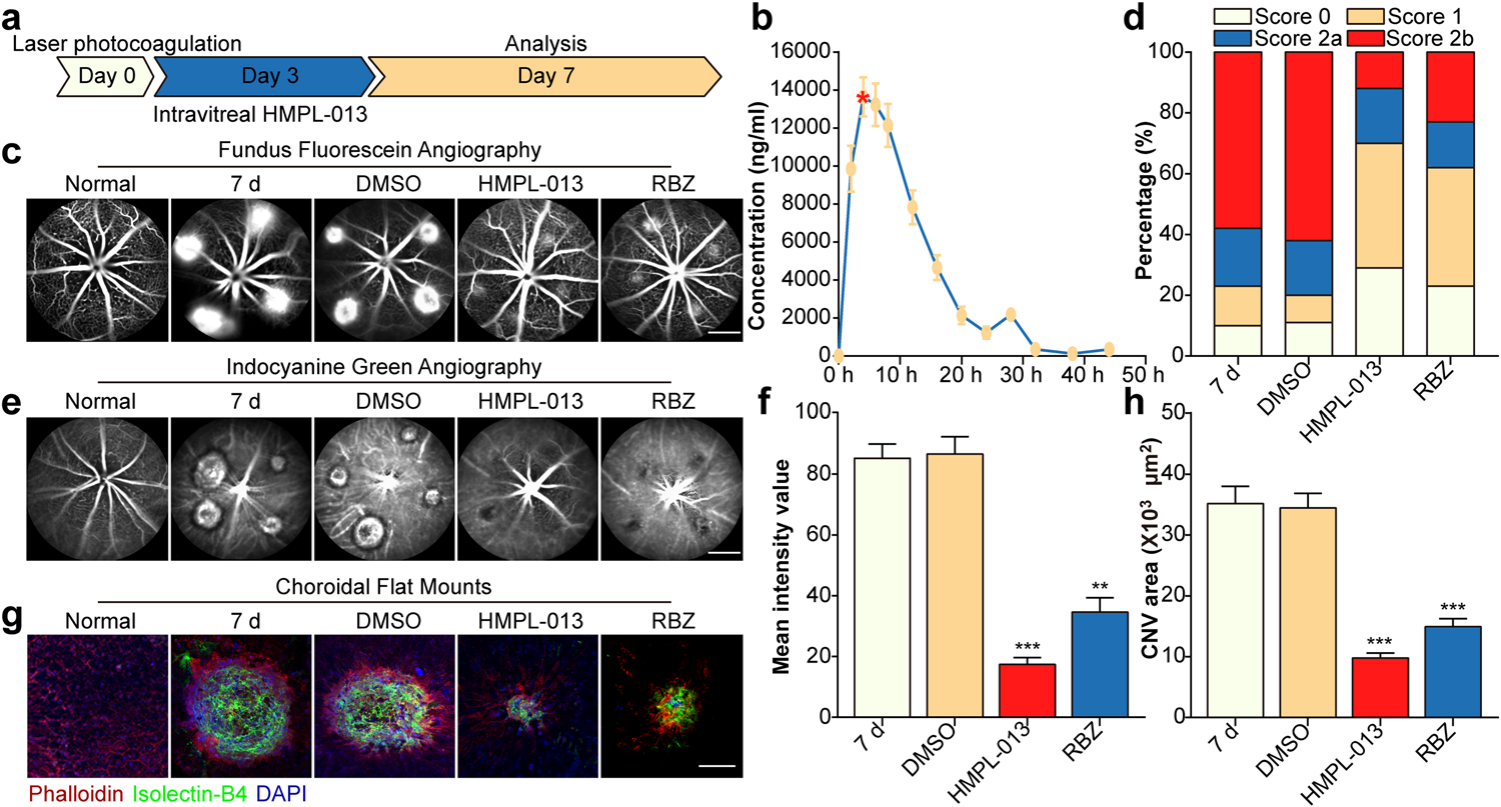
HMPL-013 mitigates mouse laser-induced CNV formation. **a** Schematic of experiment design. Diagram of schedule for CNV induction (D0), fruquintinib (HMPL-013) intravitreal injection (D3), and FFA, ICGA, and IF analysis (D7). **b** Mean retinal/choroidal/scleral concentration–time profiles of HMPL-013 in mice after intravitreal injection of [^14^C] HMPL-013. The mice were randomly assigned into five groups: normal, CNV 7 d, CNV 7d + DMSO, CNV 7 d + HMPL-013, and CNV 7 d + ranibizumab (RBZ). **c** FFA was performed in each group. **d** FFA of CNV lesion and fluorescein leakage in CNV lesions was graded in CNV 7 d, CNV 7 d + DMSO, CNV 7 d + HMPL-013, and CNV 7 d + RBZ groups. **e** ICGA was performed in each group. **f** Mean intensity value representing CNV leakage in FFA was quantified. ^**^P < 0.01, ^***^P < 0.005 vs. CNV 7 d group. **g** Representative image of choroid flat mounts after triple staining with FITC-IB4 (green), rhodamine-phalloidin (red), and DAPI (blue). **h** The CNV area was analyzed. ^***^P < 0.005 vs. CNV 7 d group. *n* = 5 in each group.

To explore the effects of HMPL-013 on mouse CNV formation, HMPL-013 intravitreal injection was done at day 3 after laser coagulation, and analysis was done at day 7 (Fig. 1a). The concentration–time profiles of HMPL-013 in the mouse retina–RPE–choroid complex tissues after [^14^C] HMPL-013 intravitreal injection showed that HMPL-013 reached its peak concentration at 4 h (13649.21 ng/ml ± 1024.80 ng/ml) and the high concentration sustained until 20 h (Fig. 1b). CNV leakage was alleviated in the HMPL-013 and RBZ groups compared to the CNV 7 d group (Fig. 1c). The leakage score analysis further showed that the percentage of 0 and 1 scores increased, while the percentages of 2a and 2b scores decreased, in the HMPL-013 and RBZ groups compared to the CNV 7 d group (Fig. 1d). CNV area also decreased in HMPL-013 and RBZ groups (Fig. 1e). Accordingly, the mean intensity values showed that CNV leakage decreased in the HMPL-013 and RBZ groups (Fig. 1f). In addition, staining of IB4 (a vascular endothelial cell marker) and phalloidin was performed on choroidal flat mounts, indicating that HMPL-013 alleviated CNV formation (Fig. 1g, h). The results suggested that HMPL-013 intravitreal injection reduced CNV leakage and area.

### HMPL-013 causes no obvious intraocular toxicity

**Fig. 2:**
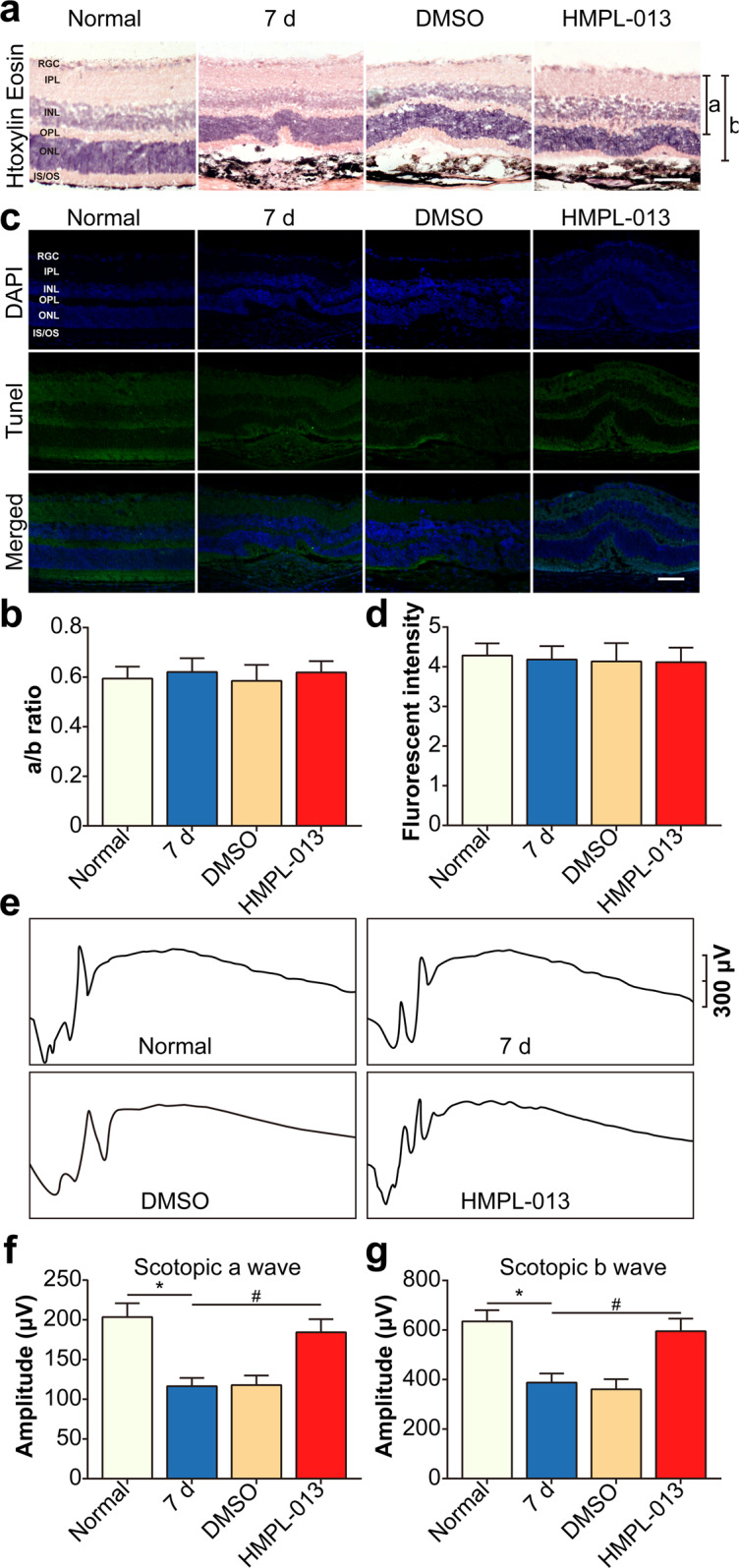
HMPL-013 causes no intraocular toxicity. The mice were randomly divided into four groups:normal, CNV 7 d, CNV 7 d + DMSO, and CNV 7 d + HMPL-013. **a** HE staining on retina–RPE–choroid complex paraffin section was performed. **b** Quantification of the ratio of A to B was shown. **c** DAPI (blue) and TUNEL (green) stain on retina–RPE–choroid complex cryosections was performed. **d** The TUNEL fluorescent intensity was analyzed. **e** Representative scotopic ERG tracings at the light intensity of −1.02 log cds/m^2^. Amplitudes of **f** a-waves and **g** b-waves from normal, CNV 7 d, CNV 7 d + DMSO, and CNV 7 d + HMPL-013 groups. *n* = 3 in each group.

Next, on the basis of the efficacy of HMPL-013 on the mouse CNV, we wondered whether HMPL-013 could exert ocular side effects. HE stain on retinal cryosections (Fig. 2a) and quantification of retinal thickness (Fig. 2b) revealed no differences in histologic morphology or retinal thickness between the normal and HMPL-013-injected eyes. In addition, ocular cell apoptosis was unchanged in the HMPL-013 group compared to the normal and CNV 7 d groups (Fig. 2c, d). In CNV 7 d group, a-wave and b-wave amplitudes decreased compared to normal group, while HMPL-013 elevated a-wave and b-wave amplitudes (Fig. 2e–g), indicating that HMPL-013 improved scotopic response in mice with CNV. These data suggested that HMPL-013 intravitreal injection caused no obvious intraocular toxicity.

### HMPL-013 inhibits CNV-induced VEGF and VEGFR2 binding in choroidal vascular endothelial cells and macrophages

**Fig. 3:**
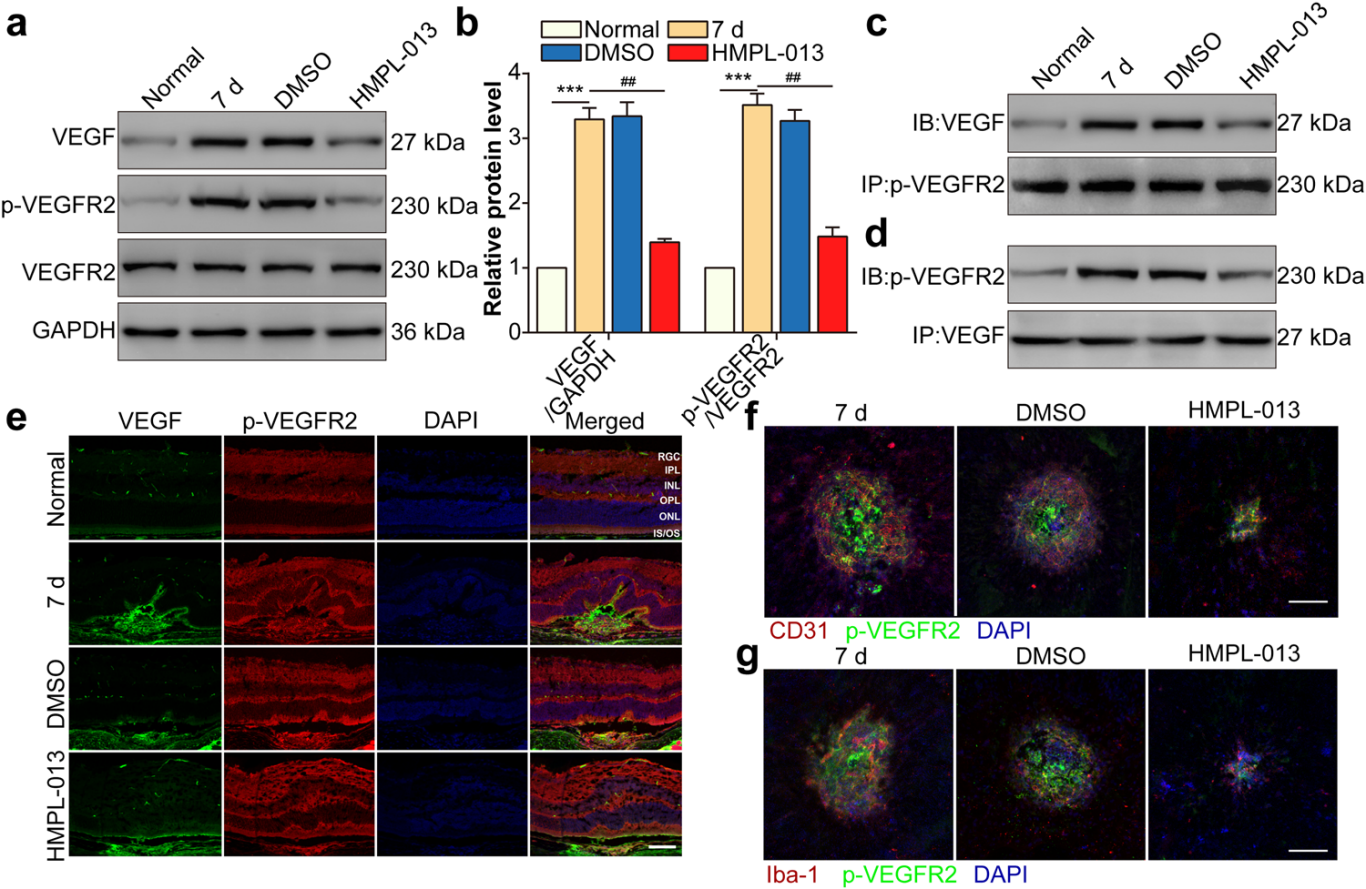
HMPL-013 inhibits CNV-induced VEGF and VEGFR2 binding in choroidal vascular endothelial cells and macrophages. The mice were randomly divided into four group: normal, CNV 7 d, CNV 7 d + DMSO, and CNV 7 d + HMPL-013. **a** Western blot was done to detect VEGF, p-VEGFR2, and VEGFR2 in retina–RPE–choroid complex tissues. **b** Densitometry values of VEGF and VEGFR2 normalized to GAPDH. ^***^P < 0.005 vs. normal group; ^##^*P* < 0.01 vs. CNV 7 d group. **c** Co-immunoprecipitation (Co-IP) was done to detect the interaction between VEGF and p-VEGFR2 in retina–RPE–choroid complex tissues using anti-p-VEGFR2 antibody as the bait. **d** Co-IP was done to detect the interaction between VEGF and p-VEGFR2 in retina–RPE–choroid complex tissues using anti-VEGF antibody as the bait. **e** Immunostaining of p-VEGFR2 (red), VEGF (green), and DAPI (blue) on retina–RPE–choroid cryosections. **f** Immunostaining of CD31 (endothelial cell marker, red), p-VEGFR2 (blue), and DAPI (blue) on choroidal flat mounts. **g** Immunostaining of IBA-1 (macrophage marker, red), p-VEGFR2 (blue), and DAPI (blue) on choroidal flat mounts. *n* = 10 in each group.

We have found HMPL-013 alleviated CNV formation without ocular toxicity. Therefore, the question of how did HMPL-013 play a therapeutic role during CNV arose. Laser-induced VEGF and p-VEGFR2 protein level in retina–RPE–choroid complex was downregulated by HMPL-013 (Fig. 3a, b). Moreover, laser-induced interaction between VEGF and p-VEGFR2 was inhibited by HMPL-013 (Fig. 3c, d). Immunostaining also showed that the colocalization of VEGF and p-VEGFR2 was decreased by HMPL-013 (Fig. 3e). Furthermore, the colocalization between CD31 (an endothelial cell marker) and p-VEGFR2 (Fig. 3f), as well as Iba-1 (a macrophage marker) and p-VEGFR2 (Fig. 3g), was suppressed by HMPL-013. The data suggested that HMPL-013 might inhibit CNV-induced VEGF and p-VEGFR2 binding in choroidal vascular endothelial cells and macrophages.

### HMPL-013 alleviates CNV-induced macrophage M1 polarization

**Fig. 4:**
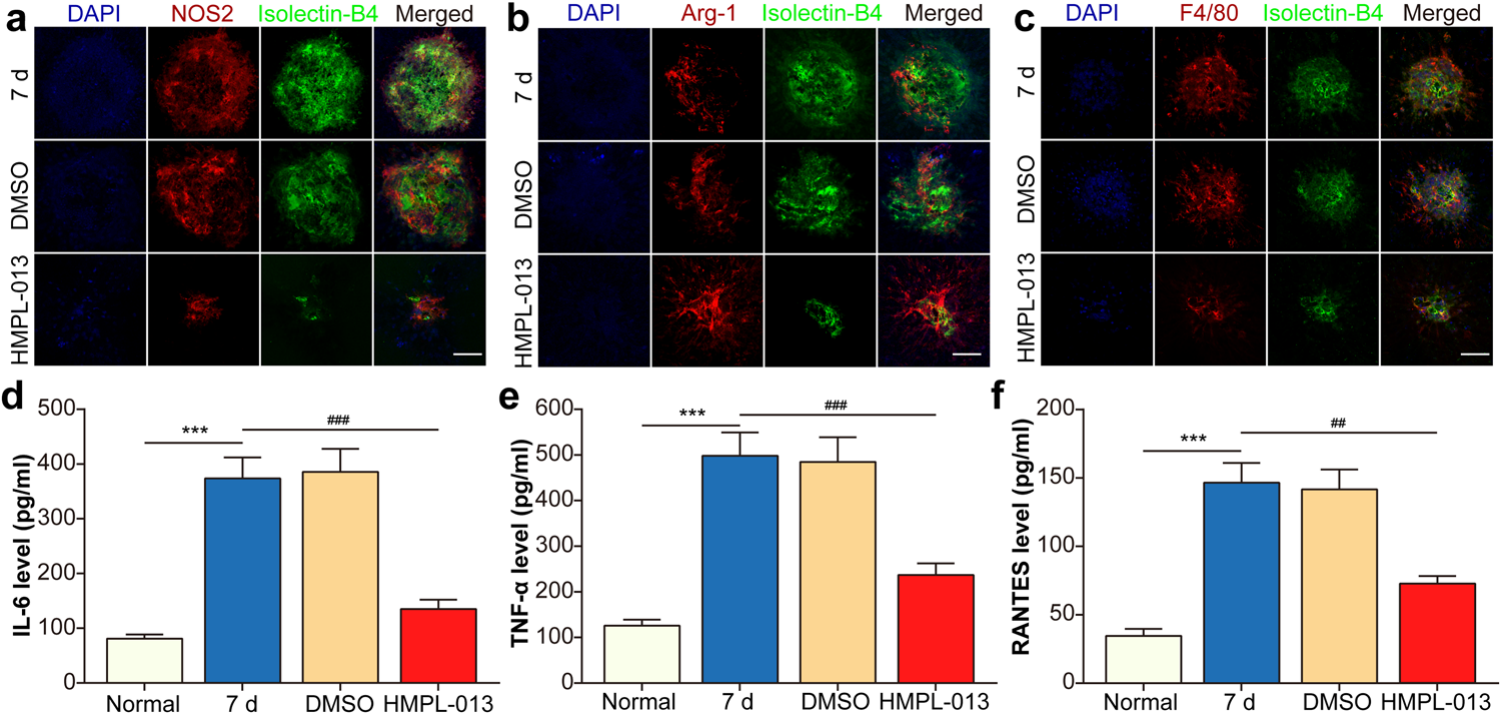
HMPL-013 alleviates CNV-induced macrophage M1 polarization. The mice were randomly divided into four groups: normal, CNV 7 d, CNV 7 d + DMSO, and CNV 7 d + HMPL-013. **a** Immunostaining of NOS2 (M1-type macrophage marker, red), IB4 (green), and DAPI (blue) on choroidal flat mounts. **b** Immunostaining of Arg1 (M2-type macrophage marker, red), IB4 (green), and DAPI (blue) on choroidal flat mounts. **c** The immunofluorescence of DAPI (blue), F4/80 (red), and IB4 (green) was shown. ELISA was done to detect pro-inflammatory cytokines, including IL-6 (**d**), TNF-α (**e**), and RANTES (**f**) in retina–RPE–choroid complex tissues. ^***^P < 0.005 vs. normal group; ^###^*P* < 0.005, ^##^*P* < 0.01 vs. CNV 7 d group in **c**–**e**. *n* = 10 in each group.

HMPL-013 inhibited VEGF and VEGFR2 binding in macrophages infiltrating inside the CNV region. Then what was the effect of HMPL-013 on macrophage polarization? CNV-induced NOS2 (a M1-type macrophage marker) expression was inhibited (Fig. 4a), while Arg1 (a M2-type macrophage marker) expression was unaffected by HMPL-013 (Fig. 4b). Moreover, HMPL-013 downregulated macrophage infiltration induced by CNV (Fig. 4c). Meanwhile, pro-inflammatory cytokines produced by M1-type macrophages, including IL-6 (Fig. 4c), TNF-α (Fig. 4d), and RANTES (Fig. 4e), increasing in the CNV 7 d group, was downregulated by HMPL-013. The results suggested that HMPL-013 inhibited CNV-induced macrophage M1 polarization.

### HMPL-013 downregulates hypoxia-induced VEGF/VEGFR2/PI3K/AKT/NF-κB pathway and CCL2 secretion in HCVECs

**Fig. 5:**
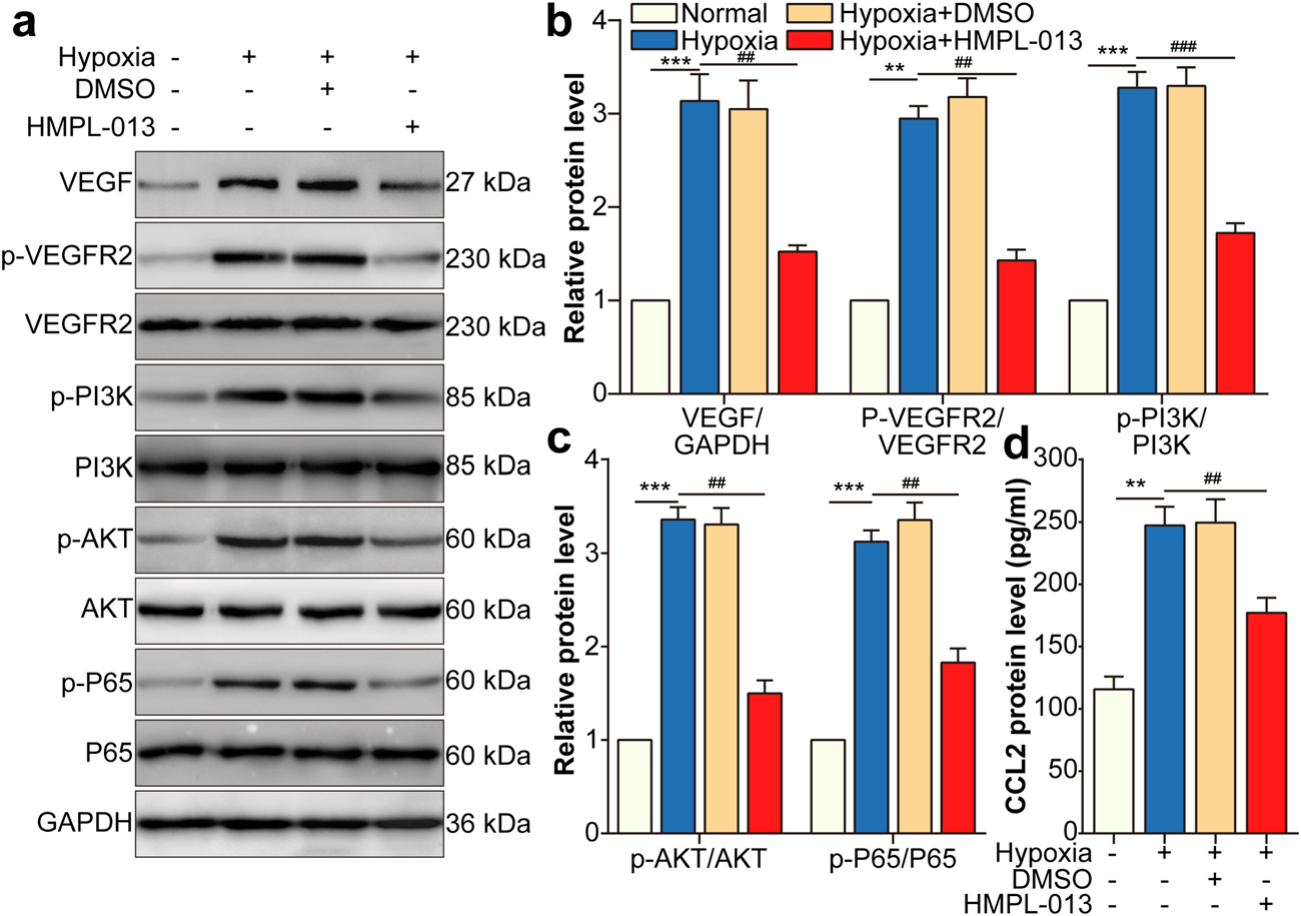
HMPL-013 downregulates hypoxia-induced VEGF/VEGFR2/PI3K/AKT/NF-κB pathway and CCL2 secretion in HCVECs. HCVECs were randomly assigned into four groups: normal, hypoxia (24 h), hypoxia + DMSO, and hypoxia + HMPL-013 (0.05 μmol/l for 24 h). **a** Western blot was done to detect VEGF, p-VEGFR2, VEGFR2, PI3K, p-AKT, AKT, p-p65, and p65 protein level. **b** Densitometry values of VEGF normalized to GAPDH, p-VEGFR2 normalized to VEGFR2, and p-PI3K normalized to PI3K. ^***^P < 0.005, ^**^P < 0.01 vs. normal group; ^###^*P* < 0.005, ^##^*P* < 0.01 vs. CNV 7 d group. **c** Densitometry values of p-AKT normalized to AKT, and p-P65 normalized to P65. ^***^P < 0.005 vs. normal group; ^##^*P* < 0.01 vs. CNV 7 d group. **d** ELISA was done to detect CCL2 protein level in culture supernatant. **P < 0.01 vs. normal group; ^##^*P* < 0.01 vs. CNV 7 d group. *n* = 4 in each group.

To further investigate the cellular and molecular mechanisms of HMPL-013 on CNV, HCVECs were cultured under hypoxia condition. Overexpression of cellular repressor of E1A-stimulated genes (CREG) attenuates atherosclerotic endothelium apoptosis via activating VEGF/PI3K/AKT pathway^23^. Moreover, an indole-3-carbinol-derived pleotropic agent OSU-A9 inhibits angiogenesis in human umbilical vein endothelial cells via partially disrupting AKT-NF-κB signaling pathway^24^. Thus, we speculated that VEGF/p-VEGFR2 binding activated downstream PI3K/AKT/ NF-κB signaling pathway. As expected, VEGF, p-VEGFR2, p-PI3K, p-AKT, and p-P65 protein level was induced by hypoxia, downregulated by HMPL-013 (Fig. 5a–c). In addition, CCL2 secretion from HCVECs was enhanced by hypoxia and inhibited by HMPL-013 treatment (Fig. 5d). The results suggested that HMPL-013 restrained hypoxia-induced VEGF/VEGFR2/PI3K/AKT/NF-κB pathway and CCL2 secretion in HCVECs.

### HMPL-013 downregulates VEGF/VEGFR2-induced macrophage M1 polarization under hypoxia condition

**Fig. 6:**
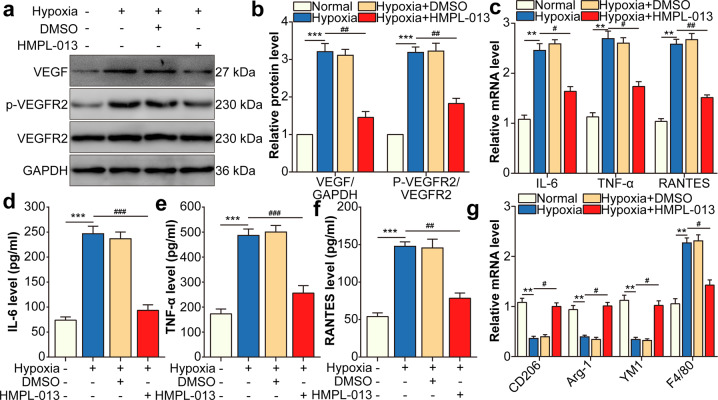
HMPL-013 downregulates VEGF/VEGFR2-induced macrophage M1 polarization under hypoxia condition. Macrophages were randomly divided into four groups: normal, hypoxia, hypoxia + DMSO, and hypoxia + HMPL-013. **a** Western blot was done to detect VEGF, p-VEGFR2, and VEGFR2 protein level. **b** Densitometry values of VEGF normalized to GAPDH, and p-VEGFR2 normalized to VEGFR2. ^***^P < 0.005 vs. normal group; ^##^*P* < 0.01 vs. hypoxia group. **c** Quantitative reverse transcription-PCR (qRT-PCR) was done to detect IL-6, TNF-α, and RANTES mRNA level. ^**^P < 0.01 vs. normal group; ^#^*P* < 0.05, ^##^*P* < 0.01 vs. hypoxia group. ELISA was done to detect IL-6 (**d**), TNF-α (**e**), and RANTES (**f**) protein level in macrophage culture supernatant. ^***^P < 0.005 vs. normal group; ^###^*P* < 0.005 and ^##^*P* < 0.01 vs. hypoxia group in **d**–**f**. **g** qRT-PCR was done to detect CD206, Arg1, YM1, and F4/80 mRNA level. ^**^P < 0.01 vs. normal group; ^#^*P* < 0.05 hypoxia group. *n* = 4 in each group.

In macrophages, hypoxia-induced VEGF and VEGFR2 expression, which was downregulated by HMPL-013 (Fig. 6a, b). In addition, M1-type macrophage markers IL-6, TNF-α, and RANTES mRNA level in macrophages (Fig. 6c) and protein level in macrophage culture supernatant (Fig. 6d–f) were upregulated by hypoxia and downregulated by HMPL-013. Meanwhile, M2-type macrophage markers CD206, Arg1, and YM1 displayed the opposite tendency to that of M1-type macrophage markers. In addition, macrophage marker F4/80 was induced by hypoxia and downregulated by HMPL-013 (Fig. 6g). The results suggested that HMPL-013 inhibited VEGF/VEGFR2-induced M1-type macrophage polarization under hypoxia condition.

### HMPL-013 inhibits HCVECs derived CCL2-induced macrophage migration and M1 polarization

**Fig. 7:**
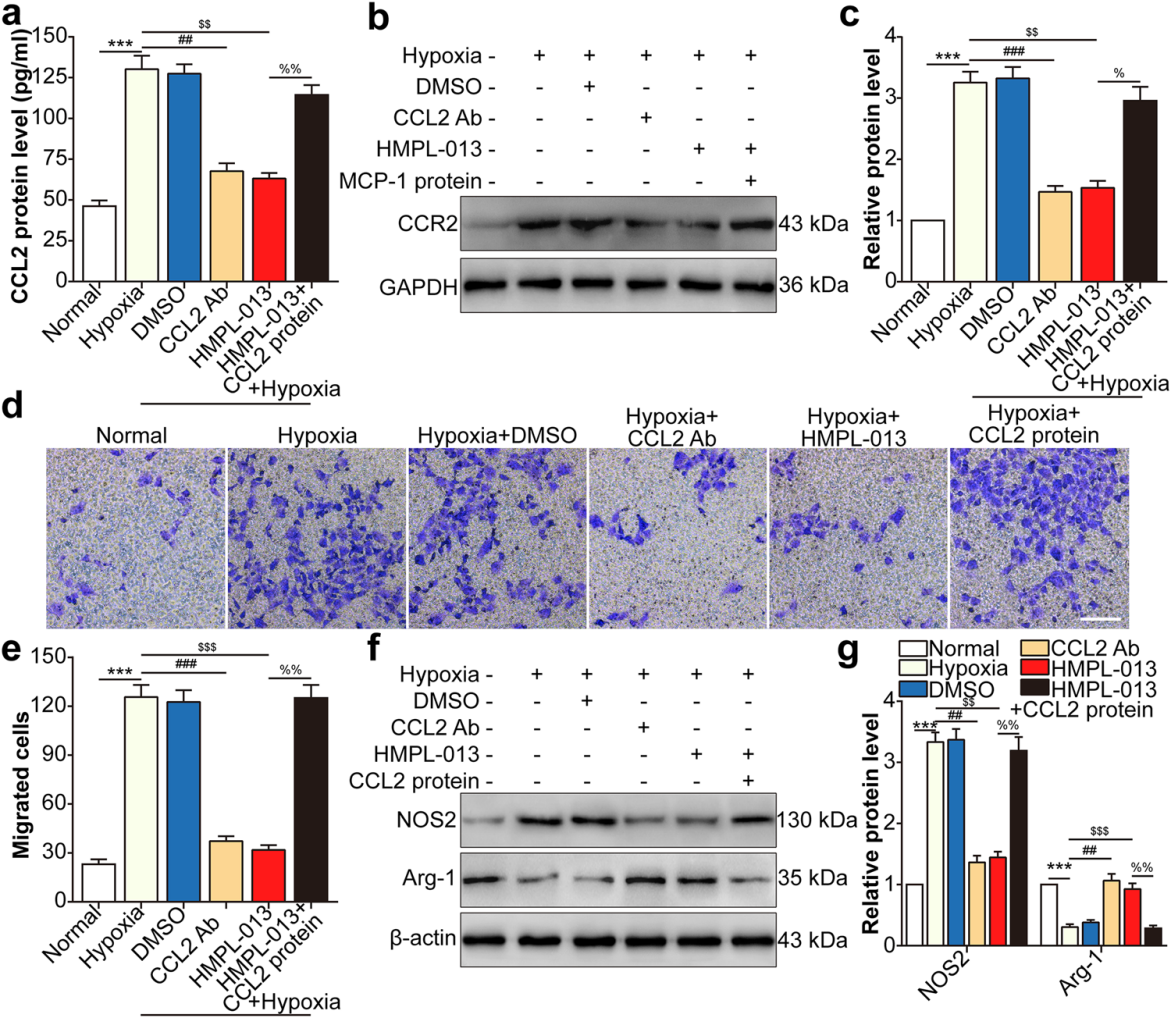
HMPL-013 inhibits HCVECs derived CCL2-induced macrophage migration and M1 polarization. HCVECs were randomly assigned into six groups: normal, hypoxia (24 h), hypoxia + DMSO, hypoxia + CCL2-neutralizing antibody (5 μg/ml for 30 min), hypoxia + HMPL-013, and hypoxia + HMPL-013 + recombinant mouse CCL2 protein (100 ng/ml for 24 h). **a** ELISA was done to detect CCL2 protein level in the macrophage culture supernatant. ^***^P < 0.005 vs. normal group; ^##^*P* < 0.01 and ^$$^*P* < 0.01 vs. hypoxia group; ^%%^*P* < 0.01 vs. hypoxia + HMPL-013 group. **b** Western blot was done to detect CCR2 protein level in the macrophage lysates. **c** Densitometry values of CCR2 normalized to GAPDH. ^***^P < 0.005 vs. normal group; ^###^*P* < 0.005 and ^$$^*P* < 0.01 vs. hypoxia group; ^%^*P* < 0.05 vs. hypoxia + HMPL-013 group. **d** Transwell assay was done to detect macrophage migration. **e** The average number of migrated macrophages was analyzed. **f** Western blot was done to detect NOS2 and Arg1 protein level in the macrophage lysates. **g** Densitometry values of each molecule normalized to GAPDH. ^***^P < 0.005 vs. normal group; ^##^*P* < 0.01, ^$$$^*P* < 0.005, and ^$$^*P* < 0.01 vs. hypoxia group; ^%%^*P* < 0.01 vs. hypoxia + HMPL-013 group. *n* = 4 in each group.

We then seek the effect of CCL2 from HCVECs on macrophage migration and polarization. HCVECs and macrophage noncontact coculture model under hypoxia condition was performed to mimic CNV microenvironment in vitro. Hypoxia promoted CCL2 in macrophage culture supernatant, which was inhibited by CCL2-neutralizing antibody or HMPL-013. CCL2 protein level was upregulated by CCL2 recombinant protein compared to hypoxia + HMPL-013 group (Fig. 7a). CCR2 protein level showed the similar tendency as that of CCL2 (Fig. 7b, c). Hypoxia facilitated macrophage migration, which was inhibited by CCL2-neutralizing antibody or HMPL-013. The inhibitory effect of HMPL-013 on cell migration was reversed by CCL2 recombinant protein (Fig. 7d, e). M1-type macrophage marker NOS2 protein level was upregulated by hypoxia, and downregulated by CCL2-neutralizing antibody or HMPL-013. The negative regulatory function of HMPL-013 was impaired by CCL2 recombinant protein. M2-type macrophage marker Arg1 protein level showed the opposite tendency to that of NOS2 (Fig. 7f, g). The results suggested that HMPL-013 inhibited HCVECs derived CCL2-induced macrophage migration and M1 polarization.

### HMPL-013 inhibits macrophage M1 polarization-induced HCVECs proliferation, migration, and tube formation

**Fig. 8:**
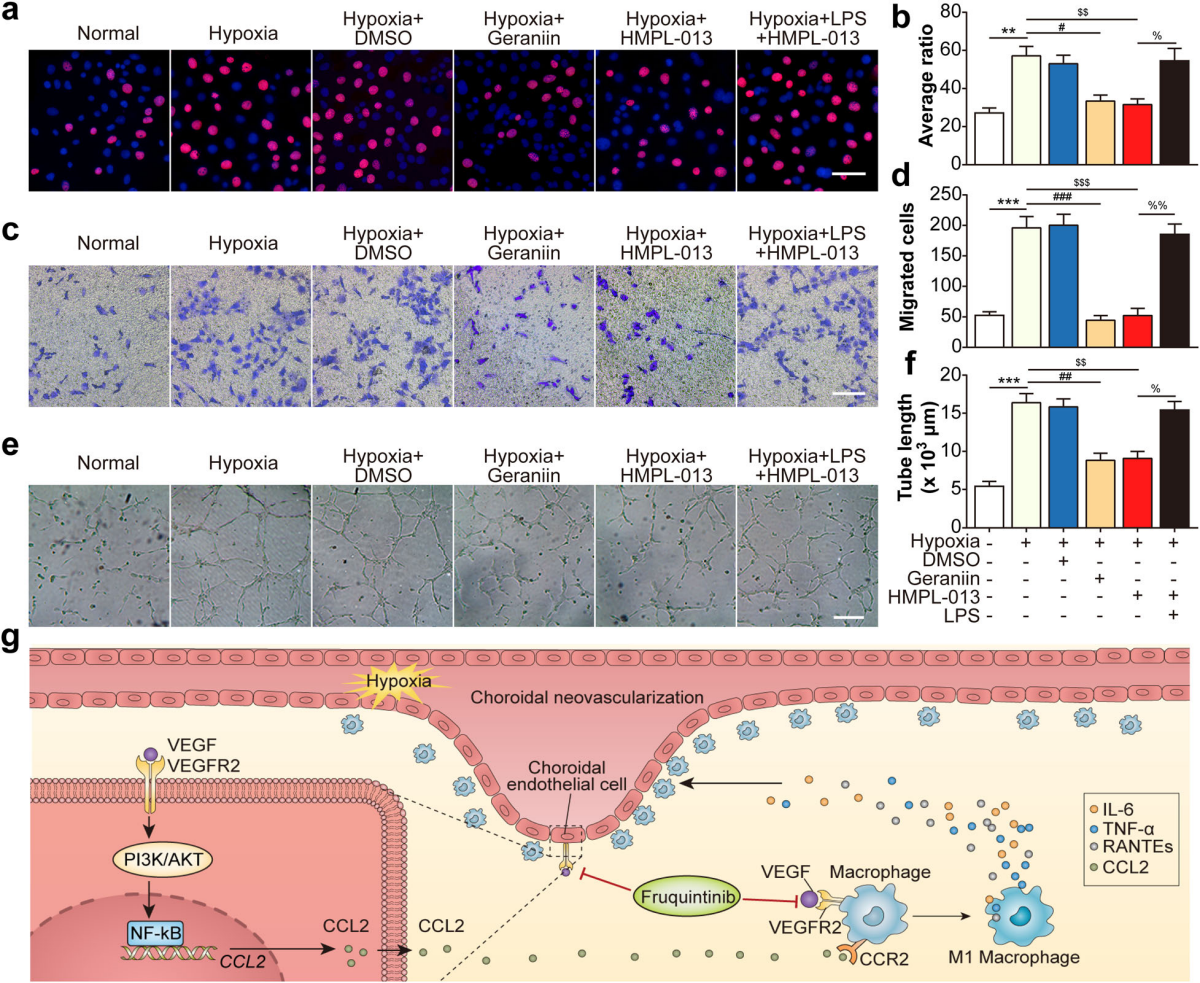
HMPL-013 inhibits macrophage M1 polarization-induced HCVECs proliferation, migration, and tube formation. Macrophages were randomly assigned into normal, hypoxia, hypoxia + DMSO, hypoxia + geraniin (30 μM for the last 2 h), hypoxia + HMPL-013, and hypoxia + HMPL-013 + LPS (2 μg/ml for 24 h). **a** EdU assay was done to detect HCVECs proliferation. EdU (red) and DAPI (blue) were stained. **b** The average ratio of the number of EdU-positive cells compared to the number of DAPI-positive cells was analyzed. ^**^P < 0.01 vs. normal group; ^#^*P* < 0.05 and ^$$^*P* < 0.01 vs. hypoxia group; ^%^*P* < 0.05 vs. hypoxia + HMPL-013 group. **c** Transwell assay was done to detect HCVECs migration. **d** The average number of migrated HCVECs was analyzed. ^***^P < 0.005 vs. normal group; ^###^*P* < 0.005 and ^$$$^*P* < 0.005 vs. hypoxia group; ^%%^*P* < 0.01 vs. hypoxia + HMPL-013 group. **e** Tube formation assay was done in HCVECs. **f** The average number of closed tubes was analyzed. ^***^P < 0.005 vs. normal group; ^##^*P* < 0.01 and ^$$^*P* < 0.01 vs. hypoxia group; ^%^*P* < 0.05 vs. hypoxia + HMPL-013 group. *n* = 4 in each group. **g** Schematic diagram of pathways was shown. HMPL-013 inhibited VEGFR2/VEGF/PI3K/AKT/NF-κB/CCL2 pathway in choroidal vascular endothelial cells, downregulating endothelial cell CCL2 secretion to mitigate macrophage infiltration and M1-type macrophage polarization. Meanwhile, HMPL-013 inhibited VEGFR2/VEGF binding on the surface of macrophages, subsequently decreased the accumulation of macrophages inside the CNV lesion, M1 macrophage polarization, and pro-inflammatory cytokine secretion, which promoted endothelial cell proliferation, migration, and tube formation. Thus, HMPL-013 might break the detrimental cross talk between endothelial cells and macrophages to inhibit CNV formation.

Finally, we investigated the effects of pro-inflammatory cytokines derived from macrophages on the pro-angiogenic behaviors of HCVECs. HCVECs proliferation was induced by hypoxia, reduced by macrophage polarization modulator geraniin or HMPL-013. The downregulatory role of HMPL-013 on the proliferation of HCVECs was weakened by macrophage M1-type polarization agonist LPS (Fig. 8a, b). Similarly, HMPL-013 played the inhibitory role on hypoxia-induced HCVECs migration (Fig. 8c, d) and tube formation (Fig. 8e, f). The results suggested that HMPL-013 inhibited macrophage M1 polarization, consequently suppressed HCVECs proliferation, migration, and tube formation. To sum up, HMPL-013 mitigated mouse CNV formation via inhibiting VEGF/VEGFR2 binding in CECs and macrophages, consequently blocking the detrimental cross talk between these two kinds of cells (Fig. 8g).

## Discussion

Anti-VEGF drugs such as ranibizumab and bevacizumab are neutralizing monoclonal antibodies to VEGF-A^25^. Conbercept is a recombination fusion protein of VEGF receptor and human immunoglobulin Fc segment gene, functioning on multiple VEGF targets, including VEGF-A, VEGF-B, and PGF^26^. Compared to the currently used anti-VEGF drugs, HMPL-013 has an advantage of inhibiting the activation of all types of VEGFRs^27^, consequently suppressing the binding of all type VEGF family members to their receptors and downstream signaling pathways. We also found HMPL-013 at the dose of 5 μg alleviated mouse CNV leakage and area, even better than RBZ at the dose of 10 μg. To date, HMPL-013 has been progressed in clinical trial for advanced non-small cell lung cancer^28^ and advanced gastric cancer^29^. Whether HMPL-013 can be used for the treatment of CNV needs further investigation.

VEGF and its receptor VEGFR2 contribute to the progression of CNV. The interfering strategies targeting VEGF/VEGFR2 axis alleviate CNV, such as intravitreal injection of Apatinib (an inhibitor of VEGFR2; in mice)^30^ BC001 (An antibody of VEGFR2; in rhesus monkeys)^31^, and heparin (inhibits VEGFR2; in mice)^32^ further indicate VEGF/VEGFR2 axis can be a potential therapeutic target for CNV. In the study, HMPL-013 also downregulated VEGF and VEGFR2 expression, and their binding in CECs and macrophages.

Previous study reveals the M1-associated cytokines increased to a greater extent in the RPE/choroid complexes, whereas the M2-associated cytokines were highly expressed in the retinas^33^. While human advanced AMD macula has a higher M1 to M2 chemokine transcript ratio compared to normal autopsied eyes^10^. Study using transgenic mice advance inflammatory M1 phenotype monocyte (CCR2^+^) infiltrate as the drive for experimental CNV^34^. However, another study illuminates that VEGF^+^Arg1^+^ macrophages drive the onset of CNV in mice^35,36^. These contradictory results about the roles of M1 and M2 macrophages in the pathogenesis of CNV potentially due partially to the complex and kinetic microenvironment that governs macrophage polarization and function^37,38^. Hereon, we found HMPL-013-mitigated CNV formation via inhibiting macrophage M1 polarization.

The mRNA expressions of M1-related markers are dramatically upregulated in the early stage, while the M2-related markers are slightly upregulated in the middle stage and sustained until the late stage in the aqueous humors of wet AMD patients^39^. In the study, the mouse choroidal flat mounts on day 7 following laser treatment were used for the detection of macrophage polarization, showing that both of M1- and M2-type markers increased. The difference might attribute to the different species and tissues.

VEGFR1 knockdown inhibits MCP-1 (CCL2) expression of clear cell renal cell carcinoma cells^40^. It has been reported that MCP-1 (CCL2) via nuclear factor kappa β in bovine retinal endothelial cells^41^. HMPL-013 is a potent inhibitor for all kinds of VEGFRs. Therefore, we speculate that HMPL-013 inhibits the binding of VEGFs to VEGFRs in HCECs to downregulate the expression of CCL2. Thereafter, downregulated CCL2 restrain the infiltration of macrophages, resulting in the decreased expression of CCR2.

In summary, HMPL-013 ameliorated mouse CNV formation via inhibiting VEGF/VEGFR2 binding in CECs and macrophages, thereby blocking the detrimental cross talk between these two kinds of cells. However, the study still left certain questions needed for further exploration, such as the inhibitory roles of HMPL-013 on other kinds of VEGF family members, and dynamic observation for HMPL-013 treatment on the progress of mouse CNV.

## Supplementary information

Supplementary Table The sequences of primers used in the study

## References

[1] Wong WL (2014). Global prevalence of age-related macular degeneration and disease burden projection for 2020 and 2040: a systematic review and meta-analysis. Lancet Glob. Health.

[2] Li J, Zhang H, Sun P, Gu F, Liu ZL (2013). Bevacizumab vs ranibizumab for neovascular age-related macular degeneration in Chinese patients. Int J. Ophthalmol..

[3] Eichler W, Yafai Y, Wiedemann P, Reichenbach A (2004). Angiogenesis-related factors derived from retinal glial (Muller) cells in hypoxia. Neuroreport.

[4] Liu CH, Wang Z, Sun Y, Chen J (2017). Animal models of ocular angiogenesis: from development to pathologies. FASEB J..

[5] Basavarajappa HD (2017). Ferrochelatase is a therapeutic target for ocular neovascularization. EMBO Mol. Med..

[6] Gu L, Chen H, Tuo J, Gao X, Chen L (2010). Inhibition of experimental choroidal neovascularization in mice by anti-VEGFA/VEGFR2 or non-specific siRNA. Exp. Eye Res..

[7] Zhang H, He S, Spee C, Ishikawa K, Hinton DR (2015). SIRT1 mediated inhibition of VEGF/VEGFR2 signaling by Resveratrol and its relevance to choroidal neovascularization. Cytokine.

[8] Long, D. et al. VEGF/VEGFR2 blockade does not cause retinal atrophy in AMD-relevant models. *JCI Insight* **3**, e120231 (2018). 10.1172/jci.insight.120231 PMC6012504 29769445

[9] Skeie JM, Mullins RF (2009). Macrophages in neovascular age-related macular degeneration: friends or foes?. Eye.

[10] Cao X (2011). Macrophage polarization in the maculae of age-related macular degeneration: a pilot study. Pathol. Int..

[11] Zhu Y (2016). Interleukin-17A neutralization alleviated ocular neovascularization by promoting M2 and mitigating M1 macrophage polarization. Immunology.

[12] Yamada K, Sakurai E, Itaya M, Yamasaki S, Ogura Y (2007). Inhibition of laser-induced choroidal neovascularization by atorvastatin by downregulation of monocyte chemotactic protein-1 synthesis in mice. Investig. Ophthalmol. Vis. Sci..

[13] Nio Y (2012). Monocyte chemoattractant protein-1 (MCP-1) deficiency enhances alternatively activated M2 macrophages and ameliorates insulin resistance and fatty liver in lipoatrophic diabetic A-ZIP transgenic mice. Diabetologia.

[14] Li J (2018). Effect of fruquintinib vs placebo on overall survival in patients with previously treated metastatic colorectal cancer: the fresco randomized clinical trial. JAMA.

[15] Shirley M (2018). Fruquintinib: first global approval. Drugs.

[16] Shah RS (2016). Visible-light optical coherence tomography angiography for monitoring laser-induced choroidal neovascularization in mice. Investig. Ophthalmol. Vis. Sci..

[17] Zhou S (2017). A phase I study to investigate the metabolism, excretion, and pharmacokinetics of [(14)C]fruquintinib, a novel oral selective VEGFR inhibitor, in healthy Chinese male volunteers. Cancer Chemother. Pharm..

[18] Phagura RS, Suo EM, Razavi H (2020). Choroidal neovascular membrane secondary to subfoveal leukemic infiltrate. Clin. Exp. Ophthalmol..

[19] Zambarakji HJ (2006). Dose-dependent effect of pitavastatin on VEGF and angiogenesis in a mouse model of choroidal neovascularization. Investig. Ophthalmol. Vis. Sci..

[20] Guo J, Luo X, Liang J, Xiao M, Sun X (2017). Antiangiogenic effects of doxazosin on experimental choroidal neovascularization in mice. J. Ocul. Pharm. Ther..

[21] Franceschini A (2013). TNFalpha levels and macrophages expression reflect an inflammatory potential of trigeminal ganglia in a mouse model of familial hemiplegic migraine. PLoS ONE.

[22] Kelly A, Grabiec AM, Travis MA (2018). Culture of human monocyte-derived macrophages. Methods Mol. Biol..

[23] Wang N (2011). Overexpression of CREG attenuates atherosclerotic endothelium apoptosis via VEGF/PI3K/AKT pathway. Atherosclerosis.

[24] Omar HA (2013). OSU-A9 inhibits angiogenesis in human umbilical vein endothelial cells via disrupting Akt-NF-kappaB and MAPK signaling pathways. Toxicol. Appl. Pharm..

[25] Avery RL (2014). Systemic pharmacokinetics following intravitreal injections of ranibizumab, bevacizumab or aflibercept in patients with neovascular AMD. Br. J. Ophthalmol..

[26] Lu X, Sun X (2015). Profile of conbercept in the treatment of neovascular age-related macular degeneration. Drug Des. Dev. Ther..

[27] Burki TK (2018). Fruquintinib for previously treated metastatic colorectal cancer. Lancet Oncol..

[28] Lu S (2018). Randomized, double-blind, placebo-controlled, multicenter phase ii study of fruquintinib after two prior chemotherapy regimens in chinese patients with advanced nonsquamous nonsmall-cell lung cancer. J. Clin. Oncol..

[29] Chen Z, Jiang L (2019). The clinical application of fruquintinib on colorectal cancer. Expert Rev. Clin. Pharm..

[30] Kim KL, Suh W (2017). Apatinib, an inhibitor of vascular endothelial growth factor receptor 2, suppresses pathologic ocular neovascularization in mice. Investig. Ophthalmol. Vis. Sci..

[31] Zhao T (2015). Vascular endothelial growth factor receptor 2 antibody, BC001, attenuates laser-induced choroidal neovascularization in rhesus monkeys (*Macaca mulatta*). J. Ocul. Pharm. Ther..

[32] Tomida D (2011). Suppression of choroidal neovascularization and quantitative and qualitative inhibition of VEGF and CCL2 by heparin. Investig. Ophthalmol. Vis. Sci..

[33] Zhou Y (2017). Different distributions of M1 and M2 macrophages in a mouse model of laser-induced choroidal neovascularization. Mol. Med. Rep..

[34] Tsutsumi C (2003). The critical role of ocular-infiltrating macrophages in the development of choroidal neovascularization. J. Leukoc. Biol..

[35] Liu J (2013). Myeloid cells expressing VEGF and arginase-1 following uptake of damaged retinal pigment epithelium suggests potential mechanism that drives the onset of choroidal angiogenesis in mice. PLoS ONE.

[36] Xu, Y. et al. Melatonin attenuates choroidal neovascularization by regulating macrophage/microglia polarization via inhibition of RhoA/ROCK signaling pathway. *J. Pineal. Res.* **69**, e12660 (2020). 10.1111/jpi.12660 32323368

[37] Krause TA, Alex AF, Engel DR, Kurts C, Eter N (2014). VEGF-production by CCR2-dependent macrophages contributes to laser-induced choroidal neovascularization. PLoS ONE.

[38] Nagai N, Kubota S, Tsubota K, Ozawa Y (2014). Resveratrol prevents the development of choroidal neovascularization by modulating AMP-activated protein kinase in macrophages and other cell types. J. Nutr. Biochem..

[39] Yang Y (2016). Macrophage polarization in experimental and clinical choroidal neovascularization. Sci. Rep..

[40] Li C, Liu B, Dai Z, Tao Y (2011). Knockdown of VEGF receptor-1 (VEGFR-1) impairs macrophage infiltration, angiogenesis and growth of clear cell renal cell carcinoma (CRCC). Cancer Biol. Ther..

[41] Marumo T, Schini-Kerth VB, Busse R (1999). Vascular endothelial growth factor activates nuclear factor-kappaB and induces monocyte chemoattractant protein-1 in bovine retinal endothelial cells. Diabetes.

