# Supplementary material for: Fruquintinib inhibits VEGF/VEGFR2 axis of choroidal endothelial cells and M1-type macrophages to protect against mouse laser-induced choroidal neovascularization
Source: Cell Death Dis. 2020 Nov 27;11(11):1016. doi: 10.1038/s41419-020-03222-1 (PMC7695853; doi:10.1038/s41419-020-03222-1)
Supplement: Supplementary file 1 — Supplementary Table The sequences of primers used in the study [file 41419_2020_3222_MOESM1_ESM.docx]

**Supplementary Table The sequences of primers used in the study.**

| **Gene name** | **Sense sequence (5’-3’)** | **Antisense sequence (5’-3’)** |
| --- | --- | --- |
| IL-6 | GCTCCCTACTTCACAAGTCC | GCAGGTTTGCCGAGRAGATC |
| TNF-α | AGCCCACGTCGTAGCAAACCACCAA | ACACCCATTCCCTTCACAGAGCAAT |
| RANTES | TGCCCACGTCAAGGAGTATTTC | AACCCACTTCTTCTCTGGGTTG |
| CD206 | CAGGTGTGGGCTCAGGTAGT | TGTGGTGAGCTGAAAGGTGA |
| Arg1 | CTGGAACCCAGAGAGAGCAT | CTCCTCGAGGCTGTCCTTT |
| YM1 | GGGCATACCTTTATCCTGAG | CCACTGAAGTCATCCATGTC |
| F4/80 | TGTCTGAAGATTCTCAAAACATGGA | TGGAACACCACAAGAAAGTGC |
| GAPDH | CATCACTGCCACCCAGAAGACTG | ATGCCAGTGAGCTTCCCGTTCAG |
